# The Simplified Chinese Version of the Suitability Assessment of Materials for the Evaluation of Health-Related Information for Adults: Translation and Validation Study

**DOI:** 10.2196/41609

**Published:** 2023-07-07

**Authors:** Yi Shan, Meng Ji, Zhaoquan Xing, Zhaogang Dong, Ding Wang, Xiangting Cao

**Affiliations:** 1 School of Foreign Studies Nantong Univeristy Nantong China; 2 School of Languages and Cultures University of Sydney Sydney Australia; 3 Department of Urology Qilu Hospital of Shandong University Jinan China; 4 Department of Clinical Laboratory Qilu Hospital of Shandong University Jinan China

**Keywords:** simplified Chinese version of the SAM, translation, validation, suitability, health education materials

## Abstract

**Background:**

Suitable health education materials can educate people about the potential harms of high-risk factors, leading to expected behavior changes and improved health outcomes. However, most patient education materials were not suitable in terms of content, structure, design, composition, and language, as stated in the literature. There is a pressing need to use well-designed scales to assess the suitability of health education materials. Although such assessment is a common practice in English-speaking communities, few assessment tools are available in mainland China.

**Objective:**

This study aimed to translate the Suitability Assessment of Materials (SAM) for the evaluation of health-related information for adults into a simplified Chinese version (S-C-SAM) and validate its reliability for evaluating the suitability of health education materials written in simplified Chinese in mainland China.

**Methods:**

The SAM was translated into an S-C-SAM in three steps: (1) translating the SAM into an S-C-SAM, (2) translating the S-C-SAM back into an English version, and (3) testing the translation equivalence between the 2 English versions (original and back-translated) of the SAM linguistically and culturally. Any differences between these 2 English versions were resolved through a panel discussion. The validity of the S-C-SAM was determined by measuring its content validity index. The final version of the S-C-SAM was used by 3 native Chinese-speaking health educators to assess 15 air pollution–related health education materials. The Cohen κ coefficient and Cronbach α were calculated to determine the interrater agreement and internal consistency of the S-C-SAM.

**Results:**

We agreed on the final version of the S-C-SAM after settling the discrepancies between the 2 English versions (original and back-translated) and revising 2 items (sentences) rated negatively in content validation. The S-C-SAM was proven valid and reliable: the content validity index was 0.95 both in clarity and in relevance, the Cohen κ coefficient for the interrater agreement was 0.61 (*P*<.05), and Cronbach α for the internal consistency of the whole scale was .71.

**Conclusions:**

The S-C-SAM is the first simplified Chinese version of the SAM. It has been proven valid and reliable for evaluating the suitability of air pollution–related health education materials written in simplified Chinese in mainland China. It has the potential to be used for assessing the suitability of health education materials specifically selected for other health education purposes.

## Introduction

### Background

Health literacy is defined as the degree to which individuals can obtain, process, and understand basic health information and services that are needed to make appropriate health decisions [1]. Health literacy covers far more than obtaining information [2]. It emerges when health information and services seekers’ expectations, preferences, and skills meet health information and services providers’ expectations, preferences, and skills [2]. It relates to educational, social, and cultural factors impacting the individual’s expectations and preferences [2]. It involves simultaneously a more complex and interconnected set of abilities, including reading and acting on health information, communicating needs to health professionals, and understanding health instructions [3]. Nutbeam [4] proposed three subcategories of health literacy skills: (1) functional literacy: sufficient basic skills in reading and writing needed to function effectively in everyday situations; (2) communicative literacy: more advanced skills to participate in everyday activities actively, extract information, and derive meaning from different forms of communication, and apply new information to changing situations; and (3) critical literacy: more advanced skills to critically analyze information and use this information to impose better control over life events and situations. Limited health literacy influences people’s health information seeking, behavioral changes, and health decisions. In this context, patient education plays an essential role in health care [5]. After receiving education, patients are less anxious, better prepared for medical consultations, and more active in making medical decisions, therefore experiencing more positive health outcomes [6,7]. Readable and suitable education materials are effective teaching aids [8]. Such materials need to be provided to educate people about the potential harms of high-risk factors, which can lead to expected behavioral changes and desired health outcomes. To this end, the suitability of educational materials that are easy to understand and accept and readability (ie, reading difficulty) that is suitable for patient education or understanding have been recommended to improve patient knowledge [9]. However, most health education materials are produced with inadequate attention to their suitability for the intended audience [10]. As a result, they are not suitable in terms of content, structure, design, composition, and language, as stated in the literature [11].

It is imperative to provide easily accessible health information for people to improve health behaviors and health outcomes [12]. To assess the suitability of health materials for adults, Doak et al [10] developed the Suitability Assessment of Materials (SAM) for evaluation of health-related information. Although originally designed to evaluate print materials and illustrations, the SAM [12] was successfully used to assess video- and audio-taped instructions [10] and web-based materials [13]. This scale comprises a scoring sheet and instructions for evaluation criteria. There are six assessment modules consisting of 22 factors in the SAM: (1) “Content,” which consists of 3 factors; (2) “Literacy Demand,” which consists of 5 factors; (3) “Graphic Illustrations, Lists, Tables, Charts,” which consists of 5 factors; (4) “Layout and Typography,” which consists of 3 factors; (5) “Learning Stimulation & Motivation,” which consists of 3 factors; and (6) “Cultural Appropriateness,” which consists of 3 factors. The SAM was designed to rate materials on these 22 factors influencing readability and understandability. For each factor, the objective evaluation criteria provide guidance for rating materials *Superior*, *Adequate,* or *Not Suitable*, which are assigned 2 points, 1 point, and 0 points, respectively. A total score is obtained by summing the points given to the 22 factors and presented as a percentage. The percentage is calculated by dividing that sum by the total possible score. The ratings based on the obtained percentage are as follows: materials are rated *Inadequate* if they get a percentage score of 0% to 39%, *Adequate* if they get a percentage score of 40% to 69%, and *Superior* if they get a percentage score of 70% to 100%.

The SAM has been used to evaluate the suitability of particular health education materials in some studies. Robins et al [14] studied the suitability of web-based sources of information on male infertility. Sun et al [15] rated the suitability of the breast cancer treatment information disseminated on Chinese breast cancer websites. Wang et al [16] assessed the suitability of articles published by health-related WeChat public accounts. Athilingam et al [9] examined the suitability level of a mobile phone app, the Congestive Heart Failure Info App. Jawad et al [17] evaluated the suitability of the information provided on websites promoting health behaviors during infancy. Cunningham et al [18] assessed tools publicly available to parents about childhood heart failure from popular web-based venues. Cheng et al [19] evaluated the appropriateness of the information on smartphone apps that provide information about breastfeeding, formula feeding, introducing solids, or infant play for consumers. Myhre et al [20] assessed an informational website on early labor they developed. These studies provided important implications for health information providers and more specifically for effective health interventions delivered through suitable health education materials.

When there are no ready-made assessment instruments, translating existing tools into different languages is an effective and practical approach [12]. Considering that few well-developed scales are available for assessing the suitability of health education materials in Chinese-speaking societies, Chang et al [12] translated and adapted the SAM into an unsimplified Chinese (traditional Chinese) version and validated its reliability for assessing health education materials in Taiwan. To the best of our knowledge, it is the only Chinese version of the SAM, but it is an unsimplified Chinese version designed for use in Taiwan. It is necessary to develop a simplified Chinese version of the SAM (S-C-SAM) that can be used to evaluate the suitability of health education materials written in simplified Chinese in mainland China, for it is simplified Chinese (Mandarin Chinese) rather than unsimplified Chinese (traditional Chinese) that is universally used across all Chinese dialects.

### Objective

This study aimed to translate the SAM into an S-C-SAM and validate its reliability for assessing the suitability of air pollution–rated health education materials written in simplified Chinese in mainland China.

## Methods

### Translation of the SAM

Methodological issues are often involved in cross-cultural research mostly in terms of the quality of translation and the comparability of research results in different cultural and ethnic groups [21]. Literal translation does not warrant an effective questionnaire. What is more challenging is how to adapt it in a culturally relevant and comprehensible form while retaining the original meaning and intent [21]. Forward translation, back-translation, bilingual testing, and monolingual testing are essential for the translation process of study instruments, which involves cross-cultural comparisons [22]. To ensure cultural relevance and comprehensibility, we translated the SAM into an S-C-SAM following the rigorous translation procedures below.

#### Forward Translation

A native Chinese speaker translated the English SAM scoring sheet and instructions for evaluation criteria into an S-C-SAM. A qualified bilingual translator was then invited to review and identify words, phrases, sentences, and grammar that were potentially problematic or even erroneous in the S-C-SAM independently. After that, a panel discussion was held to discuss the bilingual translator’s comments and suggestions with the native Chinese translator and revise the S-C-SAM. In the discussion and revision process, great importance was attached to cultural appropriateness. It means that the core concepts in the materials correspond to the logic, language, and experiences of the target culture, and positive cultural images and examples are used in cross-cultural translation [10].

#### Back-Translation

Informed by Sperber [21], the revised S-C-SAM was translated back into English by another qualified bilingual translator who was blinded to the original English version of the SAM. To ensure the quality of back-translation, the translator was carefully selected [23].

#### Translation Equivalence Testing

We adopted Sperber et al’s [24] translation equivalence testing approach to test translation equivalence. This test was intended to validate the revised S-C-SAM by comparing the 2 English versions (original and back-translated). The comparison centered on 2 indicators: similarity of interpretability (SI) and comparability of language (CL). According to Sperber et al [24], SI means the degree to which the same responses can be produced through the 2 source-language versions despite that words used in the 2 versions are not the same, and CL points to the formal similarity between words, phrases, and sentences used in the 2 versions compared. A native English speaker was requested to make a comparison between the 2 English versions in SI and CL to find potential problems with the revised S-C-SAM. Drawing on Chang et al [12], an ordinal scale of 1 to 4 was used to rate the 2 English versions as *extremely similar*, *similar*, *not similar*, and *not at all similar* in SI, and an ordinal scale of 1 to 4 was adopted to rate the 2 English versions as *extremely comparable*, *comparable*, *not comparable*, and *not at all comparable* in CL, respectively. On the basis of the native English speaker’s ratings, we held a panel discussion to revise the corresponding problematic factors and items in the revised S-C-SAM that were given a rating of 3 or 4. The revised problematic factors and items were then retranslated for comparison with the corresponding factors and items of the original English version. This revision, retranslation, and comparison processes were repeated until the 2 English versions were comparable and interpreted nearly in the same way. Carefully considering cultural appropriateness [10], we consulted another qualified bilingual translator to further improve the S-C-SAM by revising some problematic factors and items, if any.

### Psychometric Properties Testing

Psychological properties testing involved content validation and reliability testing.

#### Content Validation

In content validation, a group of experts provide constructive feedback on the quality of the newly developed instrument and objective criteria for evaluating each item involved [25]. In this study, 3 Chinese health educators (ZD, DW, and XC) evaluated the clarity and relevance of the factors in the revised S-C-SAM to evaluate its content validity. An ordinal scale was used to rate the clarity and relevance of the final S-C-SAM: 1=*not clear*, 2=*major revisions needed to make it clear*, 3=*minor revisions needed to make it clear*, and 4=*clear*; 1=*not relevant,* 2=*major revisions needed to make it relevant,* 3=*minor revisions needed to make it relevant,* and 4=*relevant* [12]. Items rated 3 or 4 by the 3 health educators were summed, and the sum was then divided by the total number of rated items. This was the way used to measure the content validity index [25]. When negatively rated, the clarity and relevance of certain factors were further improved by a highly qualified health educator (ZX) to increase the content validity index. The final S-C-SAM was developed until this step.

All 4 health educators (ZD, ZX, DW, and XC) have a background in public health education. ZD and ZX were highly qualified health educators who have been working as professors and physicians at Qilu Hospital of Shandong University, China, since they received their doctorate at Shandong University. DW and XC are studying for their master’s degree in public health education at Shandong University. Their professional educational background and experience in engaging with patients at Qilu Hospital of Shandong University can qualify them for the content validation and reliability testing of the newly developed tool.

#### Reliability Testing

Reliability testing consisted of the testing of interrater reliability and internal consistency.

#### Interrater Reliability

We tested interrater reliability by determining the scoring consistency between independent raters. Two health educators were invited to use the final S-C-SAM to rate the suitability of 15 print air pollution–related health education materials. The Cohen κ coefficient was measured to evaluate the interrater scoring agreement. According to Fleiss [26], the Cohen κ coefficients of <0.20, 0.21-0.40, 0.41-0.60, 0.61-0.80, and >0.80 indicate poor, fair, moderate, strong, and nearly complete interrater agreement, respectively.

#### Internal Consistency

Based on 3 health educators’ ratings of a print air pollution–related health education brochure, we validated the internal consistency of the final S-C-SAM by calculating Cronbach α. We measured Cronbach α for the entire instrument with a 95% CI. A Cronbach α of ≥.70 indicated fairly acceptable internal consistency of an instrument [27,28], and a value of ≤.20 implied the removal of an item or domain [29].

### Data Collection and Analysis

We used digital scoring and rating sheets to record the collected data manually. SPSS (version 22.0; IBM Corp) was applied to analyze the data quantitatively to calculate the content validity index, Cohen κ coefficient for interrater reliability, and Cronbach α for internal consistency.

### Ethics Approval

This study was approved by the ethics review board of Qilu Hospital of Shandong University, China. The review number is KYLL-202208-026.

## Results

### Translation of the SAM

#### Forward Translation

Some problems were identified in the forward translation. The qualified bilingual translator pointed out that the wording of some translated items needed to be revised to make the S-C-SAM more linguistically and culturally appropriate or adapted although the translated version was generally understandable. That is, the translated version needed to be improved in terms of idiomatic expression through various translation techniques like addition and deletion, literal and liberal translation, and so on. For example, “the article or material” was suggested being added when some passive voice sentences were translated into active voice sentences to cater to Chinese readers’ linguistic expectations. Similarly, in the translation of “Some topics are subdivided to improve readers’ confidence” in the “Learning Stimulation & Motivation” module, “to facilitate the reader’s understanding” that functions as an adverbial of purpose should be added to improve the impressiveness and coherence of the translated sentence. Conversely, in the “Layout and Typography” module, “superior factors” should be translated into “以上情况” (“the factors above”) by both deleting “superior” and adding “above.” In the “Learning Stimulation & Motivation” module, “self-efficacy (confidence)” needed to be translated into “confidence” by deleting the technical term “self-efficacy” to avoid potential misunderstanding. Besides, some words were mistranslated. The word “images” in the “Cultural Appropriateness” module was mistranslated into “意象” (“imagery,” a literary term that means the descriptions of something such as a poem or song, and the pictures they create in mind). It should be translated into “图片” (“pictures or motifs”). In the “Learning Stimulation & Motivation” module, “Instruction models specific behavior and skills” and “Information is presented in nonspecific or category items such as food groups” needed to be further adapted linguistically, especially in terms of “models” and “specific and nonspecific” in the 2 sentences, which were proposed being translated liberally. Additionally, in “Some topics are subdivided to improve readers’ confidence” in the “Learning Stimulation & Motivation” module, “some” should not be omitted to maintain the original meaning. All these problematic aspects were revised accordingly, ensuring the correspondence of the core concepts with the logic, language, and experiences of the target culture [10], that is, the Chinese culture.

#### Back-Translation

The carefully chosen qualified bilingual translator translated the revised S-C-SAM back into English. In this process, close attention was paid to cultural appropriateness. For example, “高糖、低营养价值食物” was back-translated into “no fuel foods” rather than into “high sugar, low nutrient value foods” based on cultural differences and idiomatic expressions. In the same vein, “使用说明” was back-translated into “‘how to’ directions/instructions” to meet the native English speaker’s expectations linguistically. Syntactically, some active voice sentences were translated into passive voice sentences for concise expression and habitual passivization in popular science. These aspects of cultural appropriateness, among many others, were fully considered in the back-translation, as reported by the back-translator.

#### Translation Equivalence Testing

In the validation of translation equivalence, we also identified some problems in the back-translated modules of “Literacy Demand,” “Learning Stimulation & Motivation,” and “Cultural Appropriateness.” Most of the items in the factors included in these modules were rated 3 or 4 in SI and CL. These problematic back-translated items are presented in Table 1.

Considering that SI was crucially significant in retaining the original meaning, we kept unchanged the Chinese items in the revised S-C-SAM corresponding to those back-translated items that were rated 1 or 2 in SI, although they were rated 3 or 4 in CL in Table 1. However, we did revise the items corresponding to those back-translated items that were rated 3 or 4 in SI regardless of their ratings in CL. The revised Chinese items were then back-translated and compared with their original English items in the SAM. We repeated the revision, back-translation, and comparison process several times until the 2 English versions (back-translated and original) were judged to be interpreted nearly in the same way. Another qualified bilingual translator double-checked the revised S-C-SAM against the original English version of the SAM again. No problems were found in this final step of translation equivalence testing.

**Table 1:** Problematic back-translated items in SI^a^ and CL^b^ in comparison with corresponding items in the original English version of the SAM^c^.

Original English version	Back-translated English version	SI	CL
Common words are used all the time. Technical, concept, category, and value judgment words (CCVJ) are explained. Appropriate imagery words are used.	Everyday words are used from the beginning to the end. All professional, concept, category, and value judgment words are explained. Imagery words are used appropriately.	2	3
Common words are used frequently. Technical, CCVJ words are explained sometimes. Some jargon is used.	Many everyday words are used. Some professional, concept, category, and value judgment words are explained. Some jargon terms are used.	2	3
Uncommon words are used frequently instead of common words. No explanation or examples are given for technical and CCVJ words. Extensive jargon is used.	Many uncommon words are used. Professional, concept, category, and value judgment words are explained. Many jargon terms are used.	2	3
Nearly all topics are preceded by an advance organizer (a statement that tells what is next).	The text almost always prompts in advance before talking about a new topic (a sentence explaining what to talk about next).	2	4
Approximately 50% of topics are preceded by advance organizers.	Approximately 50% of the topics have advance reminders.	2	3
Few or no advance organizers are used.	Early hints are rarely or never used before a topic.	2	3
Instruction models specific behavior and skills. For example: nutrition information emphasizes changing eating patterns, shopping, and cooking.	Precise instructions are given for specific behaviors or skills. For example, content on nutrition emphasizes changing eating, shopping, and cooking habits.	2	3
Information is a mix of technical and common language the reader may not easily interpret in terms of daily living. For example: high sugar, low nutrient value foods instead of no fuel foods.	The article confuses professional language with everyday language, and the instructions given are not very precise, making it difficult for readers to understand. For example, “high sugar, low nutrient value foods” is used to refer to “no fuel foods.”	3	4
Information is presented in nonspecific or category items such as food groups.	No precise information is provided, such as that on grouping foods.	3	3
Central concepts of the material appear to be culturally similar to the LLE^d^ of the target culture.	The core concepts of the article are similar to the readers’ cultural concepts in cultural logic, language, and daily life.	2	3
Significant match in LLE for 50% of central concepts.	50% of the core concepts in the text are well-matched in logic, language, and daily life.	2	3
Clearly a cultural mismatch in LLE.	Logic, language, and everyday life clearly do not match the target cultural concepts.	1	3
Images and examples present culture in positive ways.	The article expresses the readers’ culture from a positive and sound perspective.	3	4
Neutral presentation of cultural images and foods.	The article presents pictures and foods of the readers’ culture in a neutral way.	3	4
Neutral presentation of cultural images and foods.	The article presents pictures and foods of the readers’ culture in a neutral way.	2	4
Negative images such as exaggerated or caricatured cultural characteristics, actions, or examples.	The text uses negative ways, such as exaggeration and satire, to show some characteristics, behaviors, or examples of the readers’ culture.	2	4

^a^SI: similarity of interpretability.

^b^CL: comparability of language.

^c^SAM: Suitability Assessment of Materials.

^d^LLE: logic, language, experience.

### Psychometric Properties Testing

#### Content Validation

Three Chinese health educators were asked to assess the content validity of the revised S-C-SAM. The revised S-C-SAM was determined to have a content validity index of 0.92 in clarity and a content validity index of 0.92 in relevance. One of the 3 health educators rated all items in all factors as 3 or 4. The other 2 health educators gave a rating of 1 or 2 to 2 Chinese items corresponding to 2 items in the SAM: “Consistently provides context before presenting new information” in clarity and “Images and examples present culture in positive ways” in relevance. Because both had a content validity index of 0.333 in clarity and relevance, these 2 Chinese items were once again revised for better clarity and better relevance. After revision, they were rated 3 or 4. The remaining 5 Chinese items rated 1 or 2 were not further revised because they were thus rated only by 1 health educator. Their content validity index of 0.666 indicated acceptable relevance and clarity. As a result, the content validity index of the final S-C-SAM was 0.95.

#### Reliability Testing

##### Interrater Reliability

Two health educator raters assessed 15 air pollution–related health education materials independently using the final S-C-SAM. The Cohen κ coefficient for the interrater agreement was determined at 0.61 (*P*<.05). Based on the measurement of interrater reliability proposed by Fleiss [26], this coefficient represented a strong interrater agreement.

##### Internal Consistency

The Cronbach α for the whole scale was determined at .71, which indicates an acceptable internal consistency of the final S-C-SAM, according to previous studies [27-29].

## Discussion

### Principal Findings

#### Translation

It is challenging to adapt a scale in a culturally relevant and understandable form while preserving its original meaning [24]. This study thus adopted forward translation, back-translation, and translation equivalence testing to ensure the cultural appropriateness of the S-C-SAM. Quality translation was warranted through 4 major quality control strategies, including forward and back-translation by 2 independent native Chinese speakers, the review of the forward-translated Chinese version by a qualified bilingual translator, the checking of the SI and CL of the back-translated English version against the original English version by a native English speaker, and the double-checking of the S-C-SAM against the SAM by a qualified bilingual translator. The problems identified and resolved in the translation stage aligned with the relevant findings by Capitulo et al [33], which were discussed in the subsection of Comparison With Previous Studies.

#### Content Validation

The content validity testing revealed that the S-C-SAM was valid, achieving a satisfactory content validity index of 0.92, although 2 items were rated 1 or 2 by 2 of the 3 health educators. After the improvement of these 2 items in clarity and relevance, the final S-C-SAM was more valid in content with a content validity index of 0.95 in clarity and relevance. This validity level was discussed in the subsection of Comparison With Previous Studies.

#### Reliability Testing

The values determined for the Cohen κ coefficient for interrater agreement (0.61, *P*<.05) and Cronbach α for internal consistency (.71) indicated a fair rating agreement between the 3 raters and an acceptable internal consistency of the final S-C-SAM, respectively. These reliability indicators were discussed in the subsection of Comparison With Previous Studies.

### Comparison With Previous Studies

It is crucial to record the methods used to translate a scale and test translation equivalence [23]. In a variety of methods adopted for translating assessment instruments [18], finding qualified translators is the first step in the translation process [12]. Skill, knowledge, and experience are all called for in the translation process [21]. Critical translation issues adversely influence many studies, even when professional translators are invited, according to Brislin [34]. This is mainly attributed to three factors: (1) some translators’ inadequate awareness of the rigorous translation requirements for cross-cultural studies; (2) their literal translation and insufficient emphasis on cultural nuances; and (3) challenges posed by colloquial expressions, slang and jargon, idiomatic phrases, and emotionally evocative words [21]. Moreover, it is not easy to find competent bilingual translators who are familiar with the content and subject area of the instrument to be translated [12,23]. Considering all the aforementioned challenges, we requested a qualified translator to forward translate the original English SAM into an S-C-SAM. This competent bilingual of Chinese and English has been engaging in medical informatics–related translation practice and studies for more than 8 years. Taking into account cultural appropriateness in terms of the nuances between the original and target languages and cultures, and the health literacy of the target users of the newly developed tool, the forward translator achieved a high level of translation equivalence in semantic contents, pragmatic meanings, and cultural characteristics. The back-translator did equally well in rendering the revised forward-translated version into an English version, in which only 3 items were rated 3 or 4 in SI. These negative ratings were found to be possibly related to the overliberal translation resulting from the revision of the forward-translated version rather than to the competence of the back-translator. Given that translation is the most commonly used method for preparing scales for cross-cultural studies [21], the choice of translators is the prerequisite for quality translation.

Sperber [21] listed some common errors in translation, including (1) adding words or phrases that are not used in the original text, (2) deleting words or phrases that are used in the original text, (3) changing the original meaning by replacing words or phrases that are used in the original text, and (4) influencing meaning and clarity negatively with poorly used grammar and syntax. This study, however, found addition, deletion, and substitution helpful for adapting the original scale linguistically and culturally, as evidenced by the instances mentioned in the “Forward Translation” subsection of the “Results” section. In the SAM, some words or phrases, for example, “image,” “superior,” “specific,” “self-efficacy,” “chunks,” and “advanced organizers”; items presented in the form of noun phrases; and passive voice items all challenged understanding if forward-translated literally. The forward translator successfully communicated their meanings by selecting culturally equivalent words, phrases, or sentences [21]. Back-translation facilitates identifying erroneous translation [21,35]. Informed by Yu et al [35], this study implemented the back-translation strategy, minimizing the risk of problematic translation by checking the back-translated English version against the original English version of the SAM in terms of SI and CL. Therefore, we detected mistranslated words, phrases, or even sentences, further improving the quality of the revised S-C-SAM that is culturally relevant, appropriate, and understandable and maintains the original meaning and intent of the source version [21,36].

This study determined the content validity index and internal consistency of the final S-C-SAM at 0.95 and 0.71, respectively. In contrast, Chang et al [12] reported that the content validity index and internal consistency of their newly developed instrument were 0.99 and 0.91, respectively. We assumed that these disparities between the scales developed in this study and those by Chang et al [12] could be somewhat attributed to the different number of raters used (3 vs 2). Limited by the number of translation and validation studies on the SAM we retrieved in the literature (ie, only Chang et al [12]), we could not make a further comparison in content validity index and internal consistency with other relevant studies to find more factors possibly influencing these indicators.

Chang et al’s [12] study was the only translation and validation study on the SAM that we found in the literature. However, many previous studies [9,13-16,30-32] directly applied the SAM to assess the usability of health education materials. Some studies [9,13-16] did not involve interrater reliability, whereas some studies [30-32] determined interrater reliability. This study attested a Cohen κ coefficient of 0.61 (*P*<.05) compared with that of 0.25 (*P*<.05) in Chang et al’s [12]; 0.53, 0.57, and 0.64 (*P*<.05) in Vallance et al’s [30]; 0.60 to 0.85 (*P*<.05) in Wallace et al’s [31]; and 0.60 (*P*<.05) in Hoffman and Ladner’s [32] studies, respectively. Informed by Hoffman and Ladner [32], we believed that these disparities in the values of the Cohen κ coefficient were primarily caused by the raters’ varying experience in evaluating health education materials. Vallance et al [30] invited expert reviewers to rate educational print resources. Hoffman and Ladner [32] used experts in written health materials and experienced raters. Similarly, we invited health educators who were experienced in assessing health education materials in the study. As a result, we achieved a strong interrater agreement in this study. However, Chang et al [12] used raters less experienced in evaluating health education materials, contributing to a relatively lower level of interrater agreement. Wallace et al [31] did not report whether the raters they invited were experienced or not. In addition, Chang et al [12] and Hoffman and Ladner [32] found that the more subjective the rating criteria the lower the interrater agreement. Therefore, we concluded that the differences in the interrater agreement were also related to raters’ different degrees of subjectivity, as reported by Weintraub et al [37] that there was “latitude allowed in the interpretation of the criteria” that possibly led to subjectivity in rating health education materials. We found that inconsistent interrater ratings mostly occurred in the modules of “Literacy Demand,” “Learning Stimulation & Motivation,” and “Cultural Appropriateness” in this study. Our finding confirmed the finding in Chang et al’s [12] study that inconsistent ratings between raters were found especially for the factors of “Literacy Demand” and “Cultural Appropriateness.” This indicates that these assessment factors in the SAM are most likely to incur rater subjectivity. Because we cannot revise these factors in the SAM to reduce subjectivity, we propose that training programs be conducted to enrich raters’ experience in assessing health education materials to improve interrater reliability [12]. Finally, we discovered that the number of raters used did not affect the values of the Cohen κ coefficient, as indicated in Table 2.

**Table 2:** Comparison of the Cohen κ coefficients and the number of raters in different studies.

Study	Cohen κ coefficient	Raters, n
This study	0.61 (*P*<.05)	2
Chang et al [12]	0.25 (*P*<.05)	2
Vallance et al [30]	0.53 (*P*<.05), 0.57 (*P*<.05), 0.64 (*P*<.05)^a^	3
Wallace et al [31]	0.60-0.85 (*P*<.05)^b^	2
Hoffman and Ladner [32]	0.60 (*P*<.05)	2

^a^Cohen κ coefficient was calculated between each pair of the 3 raters, resulting in 3 values of the Cohen κ coefficient.

^b^A value range rather than an overall value of the Cohen κ coefficient was reported by Wallace et al [31].

### Limitations

This study has some limitations. First, all possible linguistic and cultural differences could not be eliminated in the translation process, although rigorous steps were taken to ensure equivalent translation, linguistic and cultural adaptation, and effective validation. Second, we used the final S-C-SAM to evaluate the suitability of air pollution–related health education materials and validated its reliability only from the perspective of health educators. Future studies need to be conducted to attest to the validity of this newly developed instrument for assessing other health education materials from the perspective of patients and the public. On the basis of these studies, we can identify, refine, and reassess subjective rating criteria of the newly developed scale that may induce low levels of interrater scoring agreement. In this way, the newly developed instrument will have better applicability and generate findings with greater generalizability. Third, we compared the instrument developed in this study only with the tool developed by Chang et al [12] in content validity index and internal consistency. Therefore, we failed to identify other potential factors impacting these indicators in addition to the number of raters used. Finally, we did not assess concurrent validity because of the availability of few validated simplified Chinese assessment instruments similar to the SAM. However, we implemented other strategies, including back-translation, translation equivalence testing, content validity testing, and reliability testing, to warrant the validity and reliability of the newly developed simplified Chinese scale.

### Conclusions

Considering the unavailability of a simplified Chinese scale that can be used to evaluate the suitability of health education materials, we translated and adapted the SAM culturally and linguistically into the S-C-SAM and validated its reliability for assessing the suitability of air pollution–related health education materials written in simplified Chinese. The final S-C-SAM is the first validated simplified Chinese scale for evaluating the suitability of health education materials written in simplified Chinese in mainland China. This scale can enable health educators and providers to choose suitable health education materials to deliver health education and interventions to patients and the public. It can also allow those engaging in health education to develop user-friendly health education materials that can enhance the understandability and actionability of materials chosen for specific health education purposes. This will hopefully lead to immediate behavioral changes, desired medical actions, and improved health outcomes.

## Abbreviations

CL: comparability of language
S-C-SAM: simplified Chinese Suitability Assessment of Materials
SAM: Suitability Assessment of Materials
SI: similarity of interpretability

## Data Availability

Data are available upon reasonable request via victorsyhz@hotmail.com.
